# Development and validation of a novel single nucleotide polymorphism (SNP) panel for genetic analysis of *Blastomyces spp.* and association analysis

**DOI:** 10.1186/s12879-016-1847-x

**Published:** 2016-09-23

**Authors:** Holly M. Frost, Jennifer L. Anderson, Lynn Ivacic, Brian L. Sloss, John Embil, Jennifer K. Meece

**Affiliations:** 1 Department of Pediatrics, Marshfield Clinic, Minocqua, WI 54548 USA; 2 Marshfield Clinic Research Foundation, Marshfield Clinic, Marshfield, WI 54449 USA; 3 College of Natural Resources, University of Wisconsin-Stevens Point, Stevens Point, WI 54481 USA; 4 Health Sciences Centre, University of Manitoba, Winnipeg, Manitoba Canada

**Keywords:** *B. dermatitidis*, *B. gilchristii*, *Blastomyces*, Genotype, SNP, Microsatellite, Blastomycosis

## Abstract

**Background:**

Single nucleotide polymorphism (SNP) genotyping is increasingly being utilized for molecular typing of pathogens and is cost-effective, especially for large numbers of isolates. The goals of this study were 1) to develop and validate a SNP assay panel for genetic analysis of *Blastomyces spp.*, 2) ascertain whether microsatellite genotyping and the SNP genotyping with the developed panel resolve identical genetic groups, and 3) explore the utility of SNPs for examining phylogenetic and virulence questions in humans.

**Methods:**

Three hundred sixty unique *Blastomyces spp.* isolates previously genotyped with microsatellite markers were genotyped with the MassARRAY® SNP genotyping system (Agena Bioscience™, San Diego, CA), for a custom panel of 28 SNPs. Clinical presentation data was analyzed for association with SNP variants.

**Results:**

Three hundred twenty-three *Blastomyces spp.* isolates (90 %) were successfully genotyped by SNP analysis, with results obtained for at least 27 of 28 assays. For 99.7 % of isolates tested by both genotyping methods, microsatellite genetic group assignment correlated with species assignment based on internal transcribed spacer 2 (ITS2) genotyping, with Group 1 (Gr 1) being equivalent to *B. gilchristii* and Group 2 (Gr 2) being equivalent to *B. dermatitidis*. Thirteen isolates were genetic hybrids by one or both methods of genotyping and were difficult to assign to a particular genetic group or species. Fifteen SNP loci showed significantly different alleles in cases of pulmonary vs disseminated disease, at a *p*-value of <0.01 or less.

**Conclusions:**

This study is the largest genotyping study of *Blastomyces spp*. isolates and presents a new method for genetic analysis with which to further explore the relationship between the genetic diversity in *Blastomyces spp.* and clinical disease presentation. We demonstrated that microsatellite Gr 1 is equivalent to *B. gilchristii* and Gr 2 is equivalent to *B. dermatitidis*. We also discovered potential evidence of infrequent recombination between the two *Blastomyces spp.* Several *Blastomyces spp.* SNPs were identified as associated with dissemination or pulmonary disease presentation, but additional work is needed to examine virulence SNPs separately within *B. dermatitidis* and *B. gilchristii*.

**Electronic supplementary material:**

The online version of this article (doi:10.1186/s12879-016-1847-x) contains supplementary material, which is available to authorized users.

## Background

*Blastomyces spp.* are thermally dimorphic fungi endemic to regions of North America with sporadic cases in India, Africa, and South America [1–4]. Genetic typing of *Blastomyces spp.* isolates using microsatellite markers revealed two distinct genetic groups [5]. Later, significant associations between microsatellite genetic group and clinical disease phenotype were demonstrated in humans, with genetic Gr 1 being associated with isolated pulmonary disease and Gr 2 being associated with cases of disseminated disease [6]. More recently, multilocus sequence typing of *Blastomyces spp.* isolates has led to the proposal of a cryptic species, *B. gilchristii*, within the group historically referred to as *B. dermatitidis* [7]. In that study, 46 nucleotide polymorphisms were identified within 7 gene regions, with 12 SNPs being determined as diagnostic between *B. gilchristii* and *B. dermatitidis*. Genotyping of a small number of isolates by both methods indicates that *B. gilchristii*, the newly proposed species, may be equivalent to microsatellite genetic Group 1 with *B. dermatitidis* being equivalent to microsatellite genetic Gr 2 [7, 8]. This suggests that clinical disease variation is potentially associated with species-specific genetic diversity.

SNPs are a valuable tool for studying recombination, rearrangement, relatedness and other genetic processes. In humans, SNPs occur at approximately 1 SNP/kilobase throughout the genome and are responsible for most monogenic disorders [9]. Due to the versatility of SNPs to examine varying genetic questions, they are increasingly being utilized for molecular typing of pathogens [10–13]. SNP genotyping is easily suited to high-throughput testing which is more cost-effective than microsatellite typing and/or Sanger sequencing. In the case of *Blastomyces*, the ability to determine the species of an isolate from a small number of SNPs is particularly useful given the established associations with clinical features, such as disease dissemination. The goals of this study were 1) to develop and validate a SNP assay panel for genetic analysis of *Blastomyces spp.* isolates, 2) ascertain whether microsatellite genotyping and SNP genotyping with the developed panel resolve similar phylogenetic groups, and 3) explore the utility of SNPs for examining virulence associations in cases of human disease.

## Methods

### Isolates

Three hundred sixty unique *Blastomyces spp.* isolates previously extracted [6] and genotyped using 27 polymorphic microsatellite markers [7], were selected for this study. Only isolates with complete microsatellite typing for all 27 markers were included in the study. These included: 295 human isolates, 51 canine isolates, 8 environmental isolates, 4 feline isolates, 1 equine isolate, and 1 alpaca isolate. Twenty isolates were gifted to us from other researchers, three isolates were purchased from ATCC (26199, 18187, and 18188), and the remaining isolates were obtained as part of clinical diagnosis at Diagnostic Services of Manitoba (*n* = 28) or Marshfield Labs™ (*n* = 309). All isolates were identified as *Blastomyces spp.* using standard methods, which included culture of the mold form on brain-heart infusion agar with blood at 25 °C and conversion to the yeast form when incubated in Middlebrook 7H9 broth at 35 °C. Clinical presentation and mortality data was previously abstracted on 310 of these cases for a former study [6]. Research protocols were approved by the Marshfield Clinic Research Foundation Institutional Review Board. Waiver of informed consent was obtained for retrospective review of clinical information, specimen collection and genotyping.

### SNP assay development and genotyping

*Blastomyces spp.* isolates were genotyped with the MassARRAY® SNP genotyping system (Agena Bioscience™, San Diego, CA), for a custom panel of 28 single nucleotide polymorphisms (SNPs). To design our custom SNP assay, 21 gene regions were investigated for appropriate polymorphism targets that could be multiplexed into a single-well, high-throughput genotyping platform. The gene regions evaluated included known and potential virulence and housekeeping genes in both coding and non-coding areas. Alignments for each gene target were obtained from publicly available sources, National Center for Biotechnology Information (NCBI) GenBank (available at http://www.ncbi.nlm.nih.gov/genbank/) and the Broad Institute [14], and sequence data generated in our lab (data not shown). One hundred and eight different SNPs and insertion/deletions (INDELs) within 21 gene regions were evaluated for appropriate PCR and extension primer combinations. Allowing the design software to assemble multiple iterations of possible target combinations, a 28-plex assay was chosen that included at least one target from each of 19 gene regions (Table 1).

**Table 1:** Single nucleotide polymorphisms (SNPs) included in the genotyping panel

Gene Region	Function	Category	Variant _ Base pair location	NCBI reference
Chitinase	Carbohydrate metabolism, hyphal growth	Gene	chit_2396	XM_002629522.1
Microsatellite 1.32^a^	N/A	non-gene region	132GAx11_108	N/A^b^
BAD1/WI1	Surface adhesion, modulate host inflammatory response	Virulence gene^c^	BAD1_2556 BAD1_2850 BAD1_2869	U37772.1
ADP-ribosylation factor	Vesicular trafficking	Gene	ARF_374	AY013310.1
ADP-ribosylation factor 6 5′ UTR	N/A	Untranslated region	arf6_240	JN561794.1
histidine kinase	Morphogenesis, cell wall composition, sporulation	Virulence gene^d^	drk1_586^e^ drk1_595^e^	JN561950.1
alpha tubulin	Morphogenesis	Gene	TUB1_18 TUB1_277	JN562331.1
orotidine 5′-phosphate decarboxylase	Biosynthesis of pyrimidines	Gene	pyrF_21 pyrF_99	JN562191.1
chitin synthase	Cell Wall/exoskeleton scaffolding	Gene	chs2_203 chs2_290	JN561872.1
fatty acid desaturase	Membrane fluidity, thermotolerance	Gene	fads_622	JN562028.1
internal transcribed spacer 2 of rDNA	N/A	Gene	ITS2_19^e^	JN562151.1
urease	Ammonia production, protection from phagocytes	Gene	urease_1503	XM_002623809.1
alpha-[1, 3]-glucan synthase	Cell wall biogenesis, block host recognition	Gene	alpha1_3glucan_2360 alpha1_3glucan_2386	XM_002629303.1
beta-glucosidase	Breaks down cellulose	Gene	b-glucosidase_966 b-glucosidase_1243	XM_002621346.1
septin-1	Filament formation, scaffold, sporulation	Gene	septin1_1251	XM_002628186.1
heat shock protein	Thermotolerance	Gene	hsp_764	XM_002624824.1
DNA Lyase	DNA repair, Flanks mating locus	Gene	APN2_1016	XM_002623165.1
Acetate- coA ligase	Metabolism	Gene	CoAligase_346	XM_002626273.1
tryptophan tRNA ligase	ATP binding	Gene	trypt-lig_922	XM_002620210.1
tyrosinase	Involved in melanin synthesis	Gene	tyrosinase_759	XM_002623880.1

^a^Microsatellite locus 5, Meece et al. Applied and Environmental Microbiology 17:5123–5131

^b^Sequence not publically available

^c^Brandhorst et al. J Biol Chem 275:7925–7934 and Wüthrich et al. Med Mycol 44:41–49

^d^Nemecek et al. Science 312:583–588

^e^Diagnostic SNPs according to Brown et al. PLoS One 8:e59237

Two-Ten ng of each deoxyribonucleic acid (DNA) sample was amplified in a 5 μL reaction containing 1 U of Taq enzyme, 1X Buffer, 2.0 mM MgCl_2_, 500 μM each dNTP and 0.1 μM of each gene-specific forward and reverse primer (Additional file 1). Cycling conditions were 2 min at 94 °C followed by 45 cycles of 30 s at 94 °C, 30 s at 56 °C, 60 s at 72 °C and a final extension time of 5 min at 72 °C. After PCR amplification, shrimp alkaline phosphatase was added to the samples and incubated for 40 min at 37 °C. Extension primers, iPLEX enzyme, buffer, and termination mixture of mass-modified di-deoxynucleotide triphosphates were added to initiate the iPLEX primer extension reaction. The cycling conditions consisted of a two-step, 200 short cycle program with one loop of 5 cycles inside a loop of 40 cycles. The sample was denatured at 94 °C for 30 s, followed by 5 cycles of annealing at 52 °C for 5 s and extended at 80 °C for 5 s. The five annealing and extension cycles with the single denaturing step was repeated 40 times, for a total number of 200 annealing and extension cycles. MassEXTEND clean resin was added to each reaction to remove extraneous salts that interfere with matrix assisted laser desorption ionization time-of-flight (MALDI-TOF) analysis. Fifteen nL of the sample was transferred from the plate and spotted onto a matrix pad of the SpectroCHIP array. Genotypes were determined by mass correlations on the MALDI-TOF mass spectrometer.

### SNP panel validation

Twelve isolates (9 human isolates, 1 environmental isolate, 1 canine isolate, and 1 alpaca isolate) with previously obtained sequence data (available at NCBI, Table 1, and generated in our lab, data not shown), demonstrating known allelic diversity, were chosen for assay validation, to include examples of expected alleles at a subset of the targets. These 12 validation samples had been sequenced in-house for previous studies and 102 SNP alleles were known on these samples at the 28 loci included in the design. Comparison of previous in-house sequencing results with our iPLEX SNP genotyping was used to validate the accuracy of calls. Each validation sample was assayed in multiple batches of testing to confirm the precision and reproducibility of the genotyping results. Validation samples with missing alleles and inconsistent results were re-extracted to investigate sample quality and amplification inhibition. In addition to up-front assay validation, 11 duplicate samples were embedded in the final genotyping run, blinded to the genotyping technician.

### SNP panel analysis

*Blastomyces spp.* isolates with more than 1 missing allele or low probability call, as defined by the instrument software, (4 % missing genotype) were excluded from the SNP portion of the study, in order to avoid classification bias [15]. Species assignment of each isolate was based on SNP ITS2_19 [7]. Allele frequencies, expressed as percentages, were calculated for each SNP separately between the *Blastomyces spp.*

### Comparison of microsatellite and SNP genotyping

For both microsatellite typing and SNP typing data, haplotypes were ascertained by identifying matching samples, and subsumed to a single representative using Genetic Analysis in Excel v6.41 [16]. Genetic structure among the samples was analyzed separately for each genotyping method using principle coordinate analysis (PCoA) of the standardized covariance of the haplotypic genetic distance as performed in Genetic Analysis in Excel v6.41. All individual genotyping markers were weighted equally for analysis of both microsatellite typing and SNP typing data. The first and second principle coordinate were plotted to graphically illustrate clusters of haplotypes. For microsatellite data, the Bayesian approach of the program STRUCTURE [17] was used to predict the minimum number of genetic units or clusters within the composite data. Analysis settings included *K* (the putative number of genetic groups) ranging from 1 to 12, the use of the admixture model, correlated allele frequencies between populations, lambda of one, and the degree of admixture (alpha) was inferred from the data as advised by the software’s manual. The burn-in was set at 100,000 repetitions and the length of each iteration was 100,000 repetitions with five iterations of each *K*. The method of Evanno et al. [18] as estimated using Structure Harvester [19] was used to assess the most likely *K* given the data in conjunction with the mean and variance of the ln probability of *K*. STRUCTURE output was used to assign individual haplotypes to microsatellite genetic group. Locus-specific diversity measures of the microsatellite genetic groups included: number of alleles_,_ number of unique alleles, and the size and frequency of the most common allele. Genetic group assignment by microsatellite analysis and species assignment using the previously described diagnostic SNP included in the panel [7] were compared for correlation.

### SNP associations in human isolates

For human isolates with both SNP genotyping and clinical data available, associations between SNP and disease presentation were analyzed using a Pearson’s chi-square test, with α = 0.05.

## Results

### SNP panel validation

We observed 100 % concordance between Sanger sequencing and SNP calls on the 102 previously known alleles on the 12 isolate validation panel. Validation test results were 100 % reproducible when repeated in multiple batches, demonstrated good amplification, and revealed clear-cut genotyping results with all 28 assays. Furthermore, the 11 duplicate samples embedded in the genotyping project showed 100 % agreement for all SNP calls.

### SNP panel analysis

Three hundred twenty-three Blastomyces *spp*. isolates (90 %) were successfully genotyped by SNP analysis, with results obtained for at least 27 of 28 assays. The remaining 37 isolates were excluded from the SNP portion of the study due to no amplification (*n* = 7), multiple no call results (*n* = 20), or multiple heterozygous calls (*n* = 10). Results of SNP genotyping are shown in Table 2. The tyrosinase_759 SNP was unable to be genotyped on 9 isolates, making it the least robust assay. Three potential SNPs (chs2_290, fads_622, TUB1_277), designed from GenBank sequences, showed no sequence variation in the isolates tested and were excluded from further analysis. The remaining SNPs were bi-allelic as expected based on design sequences, with the exception of arf6_240, which we discovered to be tri-allelic. Three polymorphisms (ITS2_19, drk1_586, and drk1_595) described by Brown et al. [7] as being diagnostic between *B. dermatitidis* and *B. gilchristii* were included in the assay. We found that results from genotyping of drk1_586 and drk1_595 SNPs were not in agreement with ITS2_19, as far as species assignment, on 2 isolates in this study (BD9911 and BD0503). Alternatively, results from SNPs trypt-lig_922 and CoAligase_346 assigned all 323 isolates into *B. gilchristii* and *B. dermatitidis* consistent with ITS2_19 genotyping. Based on ITS2_19, 146 (45 %) of the isolates in this study were *B. dermatitidis* with the remaining 177 isolates (55 %) being *B. gilchristii. B. gilchristii* isolates showed low allelic diversity (frequency of ≥98 % for a single allele) at all but 1 locus, chs2_203, which demonstrated a unique allele (G) at a frequency of 21 %. The majority of the diversity at this locus (73 %) was due to 27 Canadian *B. gilchristii* isolates, which all had the G allele. Alternatively, *B. dermatitidis* isolates showed much more allelic diversity, with 7 loci exhibiting frequencies of ≤90 % for a single allele. At most SNP loci (15 of 25), the most frequent allele differed between *B. dermatitidis* and *B. gilchristii*.

**Table 2:** SNP genotyping results for 323 isolates, by *Blastomyces spp.* species (ITS2_19)

Marker	Allele	*B. gilchristii*	(%)	*B. dermatitidis*	(%)
		ITS2_19 C^a^ (*n* = 177)		ITS2_19 T^a^ (*n* = 146)	
132GAx11_108	A	176	(99)	14	[10]
	G	1	[1]	132	(90)
alpha1_3glucan_2360^b^	A	1^c^	[1]	62	(42)
	C	176	(99)	84	(58)
alpha1_3glucan_2386^b^	C	1^c^	[1]	146	(100)
	G	176	(99)	0	(0)
APN2_1016^b^	C	176	(99)	134	(92)
	T	1^c^	[1]	12	[8]
ARF_374^b^	G	176	(99)	1	[1]
	A	0	(0)	145	(99)
arf6_240	A	176	(99)	4	[3]
	G	1	[1]	133	(91)
	C	0	(0)	9	[6]
BAD1_4	A	176	(99)	1^d^	[1]
	G	1^c^	[1]	145	(99)
BAD1_8^b^	A	1^c^	[1]	145	(99)
	T	176	(99)	1^d^	[1]
BAD1_9^b^	C	176	(99)	1^d^	[1]
	G	1^c^	[1]	145	(99)
b-glucosidase_1243^b^	G	177	(100)	96	(66)
	A	0	(0)	50	(34)
b-glucosidase_966	A	175	(99)	0	(0)
	G	1^c^	[1]	145	(99)
	N/A	1	[1]	1^d^	[1]
chit_2396^b^	C	177	(100)	109	(75)
	A	0	(0)	37	(25)
chs2_203	C	140	(79)	146	(100)
	G	37^e^	[21]	0	(0)
chs2_290^f^	C	177	(100)	146	(100)
	G	0	(0)	0	(0)
CoAligase_346	G	177	(100)	0	(0)
	A	0	(0)	146	(100)
drk1_586^a^	C	176	(99)	1	[1]
	T	1	[1]	145	(99)
drk1_595^a^	A	176	(99)	1^d^	[1]
	G	1^c^	[1]	145	(99)
fads_622^f^	T	177	(100)	146	(100)
	C	0	(0)	0	(0)
hsp_764^b^	A	176	(99)	13	[9]
	C	1^c^	[1]	133	(91)
pyrF_21	A	177	(100)	140	(96)
	C	0	(0)	6	[4]
pyrF_99	C	177	(100)	144	(99)
	G	0	(0)	2	[1]
septin1_1251	A	175	(99)	17	[12]
	G	2	[1]	129	(88)
trypt-lig_922	C	177	(100)	0	(0)
	G	0	(0)	146	(100)
TUB1_18	C	177	(100)	144	(99)
	T	0	(0)	2	[1]
TUB1_277^f^	C	177	(100)	146	(100)
	G	0	(0)	0	(0)
tyrosinase_759	T	174	(98)	132	(90)
	A	0	(0)	8	[5]
	N/A	3	[2]	6	[4]
urease_1503	A	177	(100)	69	(47)
	G	0	(0)	77	(53)

^a^Published by Brown et al

^b^SNP results in amino acid change

^c^Isolate BD9911

^d^Isolate BD0503

^e^Includes 27 Canadian isolates

^f^Expected SNP based on genbank sequence

### Comparison of microsatellite and SNP genotyping

**Fig. 1:**
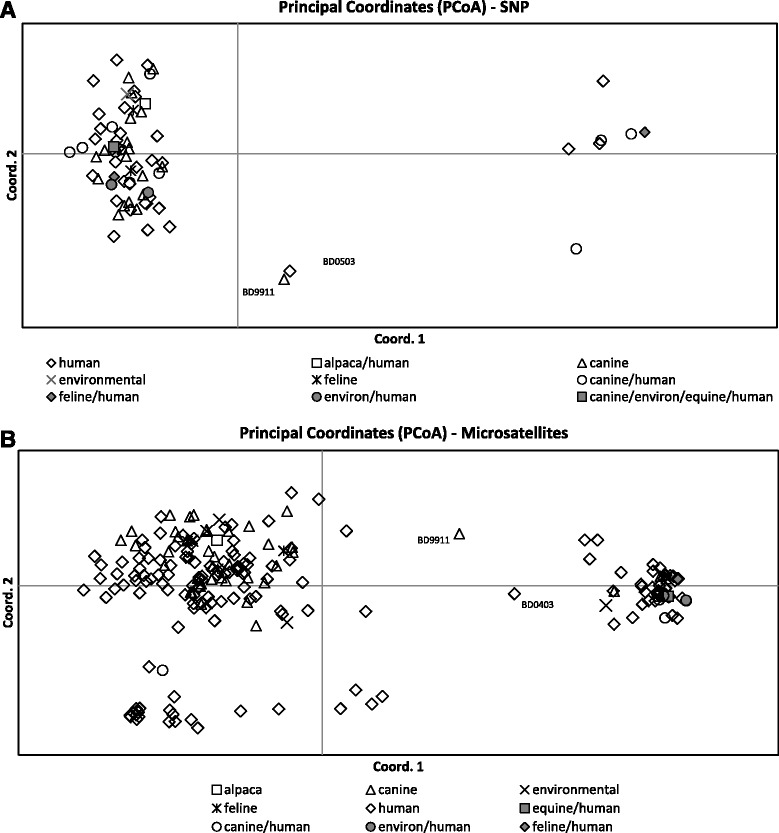
Principle coordinate analysis of the haplotypic pairwise covariance distance matrix. **a** Analysis of SNP data on 323 *Blastomyces spp.* isolates subsumed to 73 unique haplotypes. The first primary axis (Coordinate 1) explained 69 % of the variance between *B. dermatitidis* and *B. gilchristii*. **b** Analysis of microsatellite date on 360 isolates subsumed to 224 unique haplotypes. The first primary axis (Coordinate 1) explained 25 % of the variance between the genetic groups. The 3 isolates identified in the figure were genetic intermediates by 1 or both genotyping methods

The 323 *Blastomyces spp.* isolates with successful SNP genotyping were subsumed to 73 unique haplotypes (Additional file 2). SNP PCoA, shown in Fig. 1a demonstrates clustering of the haplotypes into 2 primary groups, with the exception of 2 intermediate haplotypes, isolates BD9911 and BD0503 described above. The first primary axis (Coordinate 1) explained 69 % of the variance between *B. dermatitidis* and *B. gilchristii*.

**Fig. 2:**
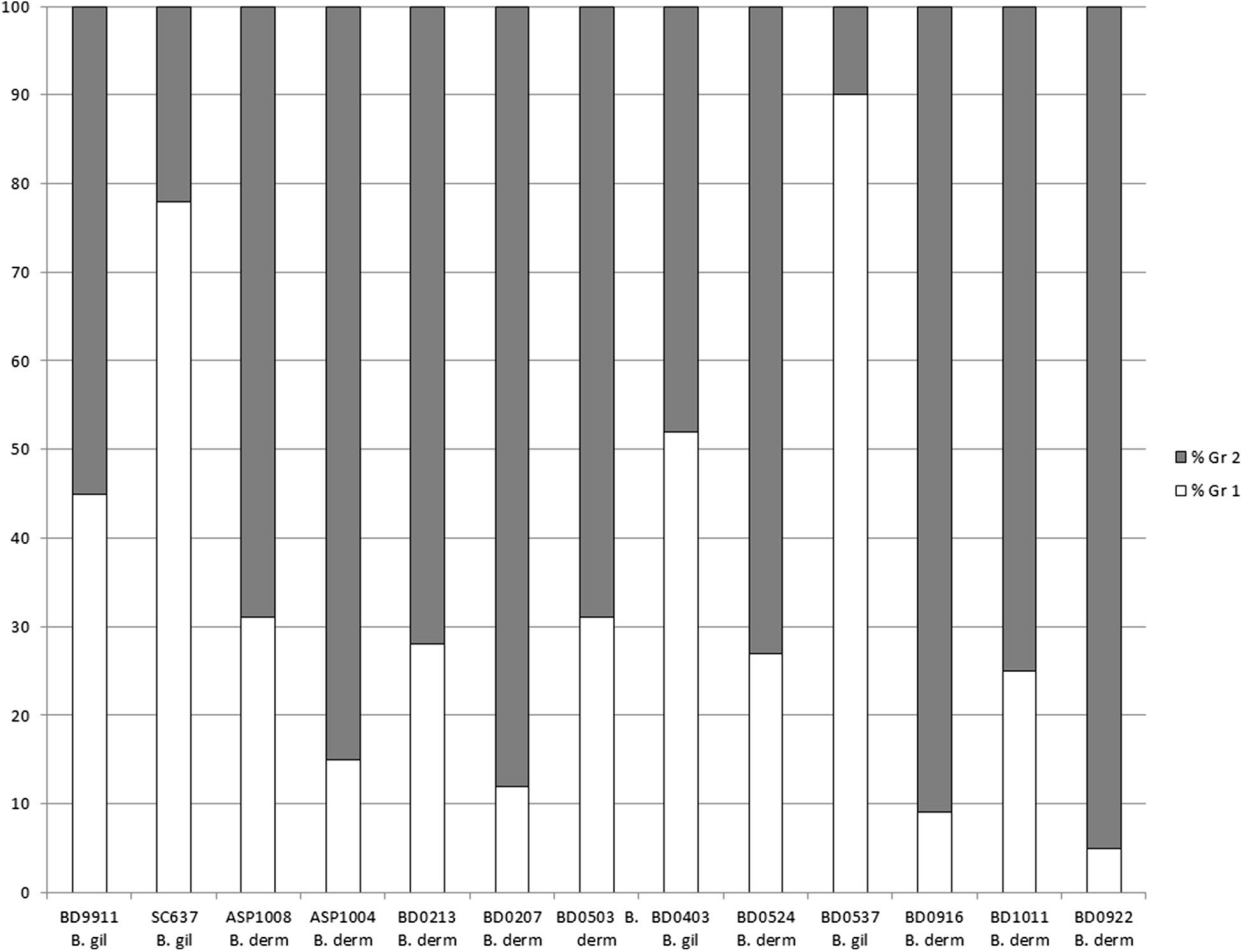
Mean *q*-values (genetic membership threshold) from microsatellite STRUCTURE analysis for 13 “hybrid” haplotypes with K = 2

Microsatellite typing data was available on all 360 isolates, including the 37 isolates that were excluded from the SNP portion of the study. Two hundred twenty-four unique microsatellite haplotypes were identified. For microsatellite PCoA, the first primary axis (Coordinate 1) explained 25 % of the variance between the genetic groups (Fig. 1b). STRUCTURE analysis of the unique microsatellite haplotypes supported two genetic units in the data (*K* = 2) based on the method of Evanno et al. [18] and the linearity and variance of lnP (D). The individual ancestry of each haplotype based on *K* = 2 revealed 62 haplotypes representing Gr 1 isolates and 162 haplotypes representing Gr 2 isolates. Examination of microsatellite group-specific allelic diversity revealed significant difference between the two genetic groups (Table 3). Gr 1 isolates show low allelic diversity, ranging from 1–7 alleles/locus (avg 3.7). Gr 2 isolates exhibit more polymorphism, ranging from 4–18 alleles/locus (avg 10.6). Across all loci, Gr 1 contained 14 alleles not present in Gr 2; Gr 2 contained 200 alleles, not represented in Gr 1. Comparison of microsatellite and SNP genotyping showed that all isolates assigned to microsatellite Gr 1 by STRUCTURE were *B. gilchristii* by ITS2_19 genotyping and all isolates assigned to microsatellite Gr 2 by STRUCTURE were *B. dermatitidis*, with the exception of BD9911. Within the data were 13 isolates with a majority q-value (genetic membership threshold) of ≤90 % (Fig. 2), by STRUCTURE analysis of microsatellite genotyping. Two intermediate isolates had nearly equal genetic membership in both groups; identified as BD9911 (genetic membership, 55 % Gr 2 and 45 % Gr 1) and BD0403 (genetic membership, 52 % Gr 1 and 48 % Gr 2).

**Table 3:** Summary of alleles by microsatellite genetic group

Group 1					Group 2			
Locus	n_A_^a^	Unique	Most Common Allele^b^	Freq^c^	n_A_^a^	Unique	Most Common Allele^b^	Freq^c^
1	3	0	250	0.73	4	1	250	0.66
2	4	0	213	0.96	9	5	203	0.34
3	5	0	199	0.93	17	12	209	0.29
4	6	0	219	0.77	10	4	229	0.40
5	3	0	214	0.98	10	7	214	0.42
6	7	3	279	0.47	9	5	265	0.58
7	4	2	273	0.56	8	6	255	0.66
8	4	0	214	0.89	12	8	208	0.49
9	4	1	194	0.93	9	6	194	0.53
10	3	0	206	0.96	10	7	206	0.55
11	4	0	155	0.98	16	12	173	0.16
12	4	0	251	0.79	10	6	249	0.23
13	4	1	192	0.98	8	5	184	0.36
14	3	0	263	0.99	9	6	269	0.38
15	3	0	198	0.99	8	5	202	0.32
16	5	0	254	0.82	15	10	258	0.49
17	3	0	232	0.98	9	6	234	0.34
18	3	0	200	0.95	18	15	224	0.15
19	4	3	231	0.94	9	8	241	0.26
20	4	2	221	0.96	13	11	235	0.34
21	3	0	189	0.92	12	9	197	0.27
22	1	0	200	1.00	13	12	206	0.23
23	4	0	220	0.98	7	3	222	0.45
24	3	0	165	0.98	8	5	167	0.53
25	3	0	250	0.98	8	5	256	0.35
26	4	2	179	0.78	14	12	167	0.44
27	3	0	235	0.59	12	9	233	0.34

^a^n_A_ = number of alleles

^b^amplicon size in base pairs

^c^Freq = frequency of the most common allele

### SNP associations in human isolates

SNP genotyping and clinical abstraction data were available on 240 human isolates. This was comprised of 151 *B. gilchristii* isolates (14 cases of disseminated disease and 137 cases of exclusively pulmonary disease) and 89 cases of *B. dermatitidis* (31 cases of disseminated disease and 58 cases of exclusively pulmonary disease). When analyzed globally without regard to species, SNP alleles were significantly different in cases of pulmonary vs disseminated disease, at a *p*-value of <0.01 or less, for the following SNPs: 132GAx11_108, alpha1_3glucan_2360, alpha1_3glucan_2386, ARF_374, BAD1_4, BAD1_8, BAD1_9, b-glucosidase_966, CoAligase_346, drk1_586, drk1_595, hsp764, ITS2_19, septin1_1251, and trypt-lig_922 (Additional file 3).

### SNPs of interest in *B. dermatitidis* and *B. gilchristii*

Seven SNP loci in *B. dermatitidis* demonstrated within species allelic diversity (frequency of ≤90 % for a single allele). Only 6 of these were located within a gene coding region. One SNP locus in *B. gilchristii* met the above criteria for diversity and was also in a coding region. These SNPs were not analyzed for association with disseminated and pulmonary disease presentation within each *Blastomyces spp.* separately due to limited statistical power. We did observe that the A urease_1503 allele (frequency 65 %) and G septin1_1251 allele (frequency 90 %) were more often observed in *B. dermatitidis* isolates resulting in cases of disseminated disease, though these SNPs do not represent amino acid changes. In addition, the A alpha1_2glucan_2360 allele, which does result in an amino acid change, showed a slightly higher frequency (52 %) in cases of disseminated disease caused by *B. dermatitidis.* In *B. gilchristii*, the G allele at locus chs2_203 was found at a higher frequency in patients with disseminated disease (43 %) as compared to pulmonary disease (18 %) and in cases resulting in death (38 %) as compared to no death (19 %). All 27 Canadian *B. gilchristii* isolates in this study exhibited the G allele at this locus.

## Discussion

This manuscript describes the development of a SNP panel for genotyping *Blastomyces spp.* isolates. SNP genotyping revealed more allelic diversity in *B. dermatitidis* isolates than *B. gilchristii* isolates, which is consistent with previous studies [7]. In *B. dermatitidis* isolates, 7 markers demonstrated a frequency of ≤90 % for a given allele in the population. *B. gilchristii* demonstrates this level of diversity in only 1 marker (chs2_203), with the remaining 27 markers showing >98 % of the population having a single allele for a given marker. A large percentage of the diversity shown in *B. gilchristii* at SNP chs2_203 is due to 27 Canadian isolates (100 % of the Canadian *B. gilchristii* isolates) that have the G allele at that locus. Only 10 additional Wisconsin *B. gilchristii* isolates (3 canine and 7 human) had the G allele at locus chs2_203 suggesting regional genetic differences. Interestingly, marker chs2_203, which demonstrates allelic diversity in *B. gilchristii,* seems to be fixed in *B. dermatitidis*.

SNP genotyping with this panel largely supports the division of *Blastomyces spp.* into 2 genetic groups, with most isolates having alleles that are characteristic of their species group. For 322/323 (99.7 %) isolates tested by both genotyping methods in this study, microsatellite genetic group assignment correlated with species assignment based on ITS2_19 genotyping, with Gr 1 being equivalent to *B. gilchristii* and Gr 2 being equivalent to *B. dermatitidis*. Several isolates in this study were particularly interesting as they appear to be genetic hybrids as determined by one or both methods of genotyping (Fig. 2). It is clear that these isolates do not fit neatly into the previously defined microsatellite or species groups and that both of these genotyping methods may be detecting hybridization between *B. dermatitidis* and *B. gilchristii*. Brown et al. [7] were only able to detect genetic recombination when it was assessed separately within each *Blastomyces sp*. This may be because only 78 samples were analyzed in that study. In this study of 323 isolates, we found evidence of potential recombination between the two species in a small percentage of isolates. In order to accurately assess genetic recombination between the two species, a larger study of more genetic intermediate isolates would be necessary.

Our SNP assay did not resolve the same level of genetic variability within *Blastomyces spp.* isolates as compared to our microsatellite assay. This is most certainly due to the higher mutation rate of microsatellite regions. In fact, previous studies for forensics applications in humans have shown that 3–4 SNPs are comparable to the genetic information in 1 microsatellite marker [20, 21]. Taking this into account, our assays were not directly comparable since they both had about the same number of loci. Furthermore the target regions of the genome were quite different between the assays with almost all of the targets included on the SNP panel being in coding regions. We must also point out that our SNP assay is multiplexed in a single low-volume reaction, whereas microsatellite typing in our lab is performed in single individual reactions for each locus. A SNP genotyping success rate of 90 % on isolates in this study can be partially explained by the fact that multiplex assays are more sensitive to factors such as degraded DNA and carryover of inhibitors, both of which we observed to a small degree in our samples. In summary, microsatellite typing had the disadvantages of being labor intensive, low throughput, and expensive, yet provides the most sensitivity for examining population genetics questions. In contrast, SNP genotyping was more cost effective, high throughput and could be used to target gene coding regions, but was less sensitive for resolving genetic differences that impact population structure. However, both methods largely discriminate the vast majority of isolates into one of the two distinct *Blastomyces spp.*

A small number of isolates, dropped from the analysis portion of the study, produced heterozygous SNP genotyping calls. In ~10 % of the DNA samples tested in this study, we observed a 260/280 ratio of <1.8 (indicating protein or phenol carry-over) or >2.0 (indicating RNA carry-over), evidence of inhibitors, and/or degraded DNA. This may be an explanation for the isolates with 1–2 “aggressive or low-probability” heterozygous calls as defined by the software. Three of the isolates had heterozygous calls for almost half of the loci, including the ITS2_19. For those isolates we propose that the patient had a dual infection, which has been previously documented in the literature by us and another group [5, 22]. It is possible that we have more isolates in our biobank that represent dual infections as we have no way of knowing how often this occurs in patients.

The discovery of heterozygous SNP calls for some of our isolates made us re-examine our hybrid isolates. None of our hybrid isolates had any heterozygous SNP calls and none of the isolates with heterozygous SNP calls were hybrids by STRUCTURE analysis. Furthermore, we verified the mating type of each of the hybrid isolates, tested previously for another study [5]. All thirteen hybrid isolates (Fig. 2) were previously tested by PCR for mating type and all were positive for only 1 allele, either the high mobility group (HMG) locus or alpha-box locus.

SNP analysis of all *Blastomyces spp.* isolates in human cases revealed significant association between SNP and disease location (exclusive pulmonary or disseminated) in 15 of the 28 loci (Additional file 3). We expected this result due to previously published associations between the divergent genetic groups of *Blastomyces spp.* and clinical features [6]. These results are included as supplementary since they represent replicated support of the already established association between genetics and virulence. In fact, among the SNPs which are significantly different between pulmonary and disseminated disease are, a SNP within a microsatellite marker previously used for the association study referenced above [6] and the *ITS2* SNP reported by Brown et al. [7] as diagnostic between *B. dermatitidis* and *B. gilchristii*.

SNP association analysis in each of the *Blastomyces spp.* separately, was unable to be performed due to limited statistical power, although several SNPs of interest were identified in *B. dermatitidis* and *B. gilchristii* for future studies. The A urease_1503, G septin1_1251, and A alpha1_2glucan_2360 alleles were more frequent in cases of disseminated disease caused by *B. dermatitidis.* In *B. gilchristii*, the G allele at locus chs2_203 was found at a higher frequency in patients with disseminated disease as compared to pulmonary disease and in cases resulting in death as compared to no death. Notably death and dissemination in *B. gilchristii* infections did not occur together frequently (only 2 of 13 cases) therefore these cases are not synonymous. It is important to point out the impact of the Canadian isolates of *B. gilchristii* on SNP allele frequency at locus chs2_203. All 27 Canadian *B. gilchristii* isolates in this study exhibited the G allele at this locus, which is represented in the United States (US) *B. gilchristii* isolates at a much smaller frequency, about 3 %. Only 17 Canadian *B. gilchristii* isolates are represented in Additional file 3, due to incomplete clinical data on some isolates. The clinical data that was available on these isolates showed a higher incidence of both disseminated disease (*n* = 5, 30 %) and death (*n* = 4, 24 %) than US isolated *B. gilchristii* cases in this study, or previously published studies [6]. This SNP association is probably not very meaningful from a virulence standpoint as it appears in a housekeeping gene and does not result in an amino acid change.

This study was limited to *Blastomyces spp.* isolates with previous microsatellite typing and only represents a limited geographic range of the organism. Additionally, SNP association analysis to clinical presentation was unable to be evaluated in *B. dermatitidis* and *B. gilchristii* separately. Despite this, the results of this study provide another tool for examining the genetic diversity of *Blastomyces spp*.

## Conclusions

This is the largest genotyping study of *Blastomyces spp*. isolates and presents a new method for genetic analysis with which to further explore the relationship between the genetic diversity in *Blastomyces spp.* and clinical disease presentation. We demonstrated that for 99.7 % of isolates tested by both genotyping methods in this study, microsatellite genetic group assignment correlated with species assignment based on ITS2_19 genotyping, with Gr 1 being equivalent to *B. gilchristii* and Gr 2 being equivalent to *B. dermatitidis*. We also discovered potential evidence of infrequent recombination between the 2 *Blastomyces spp.* In addition, several *Blastomyces spp.* SNPs were identified as associated with dissemination or pulmonary disease presentation, but additional work is needed to examine virulence SNPs separately within *B. dermatitidis* and *B. gilchristii*.

## Additional files

Additional file 1: Primers for the primary PCR amplification of each target in the 28 assay panel. List of forward and reverse primers for amplification of each SNP target. (DOCX 15 kb) Additional file 2: SNP genotyping haplotypes. Raw SNP genotyping data obtained from all isolates, subsumed to 73 unique haplotypes. (XLSX 21 kb) Additional file 3: SNP associations for disease presentation and mortality for 240 human isolates, globally for *Blastomyces spp*. Statistical analysis of SNP status by pulmonary or disseminated disease presentation. (DOCX 19 kb)

## Abbreviations

DNA: Deoxyribonucleic acid
Gr 1: Group 1
Gr 2: Group 2
HMG: High mobility group
ITS2: Internal transcribed spacer 2
NCBI: National center for biotechnology information
PCoA: Principle coordinate analysis
SNP: Single nucleotide polymorphism
US: United States
