# Supplementary material for: Development and validation of a novel single nucleotide polymorphism (SNP) panel for genetic analysis of Blastomyces spp. and association analysis
Source: BMC Infect Dis. 2016 Sep 23;16:509. doi: 10.1186/s12879-016-1847-x (PMC5035486; doi:10.1186/s12879-016-1847-x)
Supplement: Additional file 1: — Primers for the primary PCR amplification of each target in the 28 assay panel. List of forward and reverse primers for amplification of each SNP target. (DOCX 15 kb) [file 12879_2016_1847_MOESM1_ESM.docx]

| **Additional file 1. Primers for the primary PCR amplification of each target in the 28 assay panel** | | |
| --- | --- | --- |
| **Polymorphism Target** | **Forward PCR Primer sequence** | **Reverse PCR Primer sequence** |
| hsp_764 | ^1^GGAGGATGTTAAAATGCCCG | ^1^TTCGTTTCGCAATCGCTTCC |
| chit_2396 | ^1^TAAGGTCAGGAGCCACTTTC | ^1^GAACAGGGTGTGGCTCAAC |
| tyrosinase_759 | ^1^AGGGCATAGTGTTCGTGAAG | ^1^ACGGAAGGTAAAGTGTGGAG |
| chs2_203  chs2_290 | ^1^TGGGCAACAATACCTTCACC | ^1^AAGGTGCAGATGTAGCTAGG |
| trypt-lig_922 | ^1^TAAAACTTGCCCTCACGGAC | ^1^CCATCTTCTGCAATAGTGTC |
| alpha1_3glucan_2360 alpha1_3glucan_2386 | ^1^GACGCAAGTAGTGTCAAGTG | ^1^CCCTCGTAAGGTAACCAATC |
| TUB1_18 | ^1^CTCTCCGCCCAATCTTCATC | ^1^AACTGTAGCAACAAGGACCG |
| BAD1_8  BAD1_9 | ^1^AAGTGTGACTGGCACCTCTG | ^1^CCGCGTCATACTTCAAATG |
| drk1_586  drk1_595 | ^1^AAGTCAGCACTGGATCTGGG | ^1^CCCATAACAGGCATTTGGAC |
| fads_622 | ^1^TAGCCACATCCCCCAGTTTC | ^1^ACCACCTGTACCCTACAATC |
| ITS2_19 | ^1^GAACGCAGCGAAATGCGATA | ^1^ATCCGAGGTCAACCTGGTG |
| 132GAx11_108 | ^1^GCAACACCAATAAGAGAGGG | ^1^ATTCTTGTATCAGCATCCGC |
| APN2_1016 | ^1^TAAGGGCAAGGTGTTGTTGG | ^1^ATGTCTGCGGACGAGTATTC |
| TUB1_277 | ^1^CGGTCAAATTCACACGACCA | ^1^GGGTCGGCAGCTTTTCTTTC |
| b-glucosidase_966  b-glucosidase_1243 | ^1^GCCTACCCACCTGGACAGA | ^1^TTATCCAGCGCATACGCATC |
| pyrF_21  pyrF_99 | ^1^CAGACAGCTTCCCCAAAAAC | ^1^TTGATGGAGTGAGGTCGGTG |
| ARF_374 | ^1^GGGTATTTTGGAAGTAGTGC | ^1^GAAATCGTCACAACCATCCC |
| arf6_240 | ^1^CTTGTGTTTCAGCGTGTTTC | ^1^GAATCAAAACACGGCTTCGG |
| BAD1_4 | ^1^TCATTCCCTAATCGACTCGC | ^1^TACTTGTCGTCGCGAGGGTA |
| urease_1503 | ^1^AAGTTTCAGACCTGCTGCTC | ^1^TTGGGATCACTGGTAAAGGG |
| septin1_1251 | ^1^GCGGAAACGAATGTGTTGTG | ^1^CTAGCGATTTGATACCGCTG |
| CoAligase_346 | ^1^TCAAGGATCCCAACAAGGTG | ^1^AAACTTCCTTCAGGAGCTCG |
| ^1^PCR primers have a 10-mer 5' tag (ACGTTGGATG) | |  |
