# Supplementary material for: Development and validation of a novel single nucleotide polymorphism (SNP) panel for genetic analysis of Blastomyces spp. and association analysis
Source: BMC Infect Dis. 2016 Sep 23;16:509. doi: 10.1186/s12879-016-1847-x (PMC5035486; doi:10.1186/s12879-016-1847-x)
Supplement: Additional file 3: — SNP associations for disease presentation and mortality for 240 human isolates, globally for Blastomyces spp. Statistical analysis of SNP status by pulmonary or disseminated disease presentation. (DOCX 19 kb) [file 12879_2016_1847_MOESM2_ESM.docx]

| **Supplementary Table 2. SNP associations for disease presentation and mortality for 240 human isolates, globally for *Blastomyces spp*.** | | | | | | |
| --- | --- | --- | --- | --- | --- | --- |
| **Marker** | **Allele** | **Pulmonary**  (n=195) | **(%)** | **Disseminated** (n=45) | **(%)** | **p-value^1^** |
| 132GAx11_108 | A | 142 | (73) | 17 | (38) |  |
|  | G | 53 | (27) | 28 | (62) | <0.001 |
|  |  |  |  |  |  |  |
| alpha1_3glucan_2360^2^ | A | 27 | (14) | 13 | (29) |  |
|  | C | 168 | (86) | 29 | (64) | <0.01 |
|  |  |  |  |  |  |  |
| alpha1_3glucan_2386^2^ | C | 58 | (30) | 31 | (69) |  |
|  | G | 137 | (70) | 14 | (31) | <0.001 |
|  |  |  |  |  |  |  |
| ARF_374^2^ | G | 57 | (29) | 31 | (69) |  |
|  | A | 138 | (71) | 14 | (31) | <0.001 |
|  |  |  |  |  |  |  |
| BAD1_4 | A | 138 | (71) | 14 | (31) |  |
|  | G | 57 | (29) | 31 | (69) | <0.001 |
|  |  |  |  |  |  |  |
| BAD1_8^2^ | A | 57 | (29) | 31 | (69) |  |
|  | T | 138 | (71) | 14 | (31) | <0.001 |
|  |  |  |  |  |  |  |
| BAD1_9^2^ | C | 138 | (71) | 14 | (31) |  |
|  | G | 57 | (29) | 31 | (69) | <0.001 |
|  |  |  |  |  |  |  |
| b-glucosidase_966 | A | 136 | (70) | 14 | (31) |  |
|  | G | 58 | (30) | 30 | (67) | <0.001 |
|  | N/A | 1 | (1) | 1 | (2) |  |
|  |  |  |  |  |  |  |
| CoAligase_346 | G | 137 | (70) | 14 | (31) |  |
|  | A | 58 | (30) | 31 | (69) | <0.001 |
|  |  |  |  |  |  |  |
| drk1_586^3^ | C | 138 | (71) | 14 | (31) |  |
|  | T | 57 | (29) | 31 | (69) | <0.001 |
|  |  |  |  |  |  |  |
| drk1_595^3^ | A | 138 | (71) | 14 | (31) |  |
|  | G | 57 | (29) | 31 | (69) | <0.001 |
|  |  |  |  |  |  |  |
| hsp_764^2^ | A | 143 | (73) | 16 | (36) |  |
|  | C | 52 | (27) | 29 | (64) | <0.001 |
|  |  |  |  |  |  |  |
| ITS2_19 | C | 137 | (70) | 14 | (31) |  |
|  | T | 58 | (30) | 31 | (69) | <0.001 |
|  |  |  |  |  |  |  |
| septin1_1251 | A | 144 | (74) | 17 | (38 |  |
|  | G | 51 | (26) | 28 | (62) | <0.001 |
|  |  |  |  |  |  |  |
| trypt-lig_922 | C | 137 | (70) | 14 | (31) |  |
|  | G | 58 | (30) | 31 | (69) | <0.001 |
| **Marker** | **Allele** | **No Death** (n=220) | **(%)** | **Death** (n=20) | **(%)** | **p-value** |
|  |  |  |  |  |  |  |
| chs2_203 | C | 201 | (91) | 15 | (75) |  |
|  | G | 19 | (9) | 5 | (25) | <0.02^4^ |

^1^Pearson’s chi-square test with α = 0.05

^2^SNP results in amino acid change

^3^Published by Brown et al.

^4^Odds Ratio 0.28, no death is the referent group, 95% confidence interval 0.09-0.87
